# 

**Published:** 1901-06-15

**Authors:** 


### NOTICE TO CORRESPONDENTS.

All MSS., Letters, Books for Review, and other matters intended for the Editor should be addressed The Editor, "The Hospital," The Hospital Building, 28 & 29 Southampton Street, Strand, W.C.

The Editor cannot undertake to return rejected MS., even when accompanied by stamped directed envelope.

**Notice to Advertisers and Subscribers.**

All Advertisements, Orders for Copies of The Hospital, and Business Communications should be addressed to **THE MANAGER (Not to the Editor),** The Hospital, 28 & 29 Southampton Street, Strand, London, W.C.

The Cost of Subscription, post free, is as follows:—Per week, 2½d.; per quarter, 2s. 8d.; per half-year, 5s. 3d.; per annum, 10s. 6d. Foreign:—Per annum, 15s. 2d., payable, in all cases, in advance.

**NOTICE.—Vol. XXIX. of "The Hospital" (October 1900, to March, 1901), in handsome cloth binding are now on sale at the Office of this Journal, price, 7s. 6d. Cases for binding the Numbers contained in this Volume 1s. 6d. Index to Vol. XXIX., 2½d., post free.**

**The Monthly Part for June is now ready. Price 6d., by post 8d.**

### SCRAPS AND GLEANINGS.

A HOSPITAL mission ship, intended for work on the Upper Congo, has recently been built, and will shortly proceed to the Congo. The builders are Messrs. Thornycroft, of Chiswick, and the cost of the steamer was £5,630, which has been provided. A sum of £3,000 is however still required to meet the expense of sending her out. She is named the *Livingstone*.

THE sum required to build the new ward at the Diamond Jubilee Memorial Hospital at Castle Douglas, intended to be a memorial to the late Queen Victoria, is £300, of which £125 has already been raised.

A MEMORIAL TABLET of "Rose" metal has recently been placed in the Chester Infirmary in memory of the first Duke of Westminster, K.G., for 25 years President of the Infirmary.

Some Results of Sanatorium Treatment of Phthisis.

By Dr. CHOWRY MUTHU, Physician, Inglewood Sanatorium, St. Lawrence, I.W. 1/- post free.

BALE, SON & DANIELSSON, LTD., 89 Great Titchfield Street, London, W.

